# Post-intensive Care Unit COVID-19 Survivors: Functional Status and Respiratory Function Three Months After an Inpatient Rehabilitation Program

**DOI:** 10.7759/cureus.31281

**Published:** 2022-11-09

**Authors:** Ana Costa, Ana F Gonçalves, Margarida Rodrigues, Rui Santos, Miguel P Almeida, Ana Lima

**Affiliations:** 1 Physical Medicine and Rehabilitation, Centro de Reabilitação do Norte - Centro Hospitalar Vila Nova de Gaia/Espinho, Porto, PRT; 2 Spinal Cord Injuries, Centro de Reabilitação do Norte - Centro Hospitalar Vila Nova de Gaia/Espinho, Porto, PRT; 3 Physical Therapy, Centro de Reabilitação do Norte - Centro Hospitalar Vila Nova de Gaia/Espinho, Porto, PRT

**Keywords:** follow-up, disability, rehabilitation, post-intensive care syndrome, covid-19

## Abstract

Introduction and objectives

Long-term coronavirus disease 2019 (COVID-19) sequelae have become an increasing concern, with persistent dyspnoea and fatigue being the most common and long-lasting symptoms reported. The aim of this study was to evaluate the functional status and respiratory function three months after discharge from an inpatient rehabilitation program.

Materials and methods

This was a prospective study including post-ICU COVID-19 survivors consecutively admitted to an inpatient and multimodal rehabilitation program in a rehabilitation center. Evaluation of functional status (brief balance evaluation systems test (brief-BESTEST), timed up and go (TUG) test, 1 min sit to stand test (1STST), 6 min walking test (6MWT)); respiratory muscle strength (maximum expiratory pressure (MEP), maximum inspiratory pressure (MIP)); cough effectiveness (peak cough flow (PCF)); and fatigue (fatigue assessment scale (FAS)) were assessed at admission (T0), discharge (T1), and three months after discharge (T2).

Results

A total of 36 patients were included. Between T1 and T2, there was a significant improvement in MEP (84.47±20.89 vs 97.23±24.63 cmH_2_O, p<0.001), PCF (367.83±117.24 vs 441.33±132.90 L/min, p=0.003), functional capacity (1STST (19.90±6.37 vs 23.13±6.07, p=0.004), and 6MWT (459.25±153.70 vs 500.00±163.74 meters, p=0.003)). No differences were seen in MIP, brief-BESTEST, or TUG. Patients presented a higher median final FAS score at T2 compared to T1: 21.50±5 vs 18.60±2.65, p=0.002.

Conclusions

Post-ICU COVID-19 survivors admitted to an inpatient rehabilitation program maintained a good functional recovery at the three-month follow-up. Despite overall improvement, we found higher scores of FAS, suggesting worse fatigue levels.

## Introduction

Severe acute respiratory syndrome coronavirus 2 (SARS-CoV-2) is a new coronavirus responsible for a contagious disease named coronavirus disease 2019 (COVID-19), which has quickly evolved into a worldwide pandemic [1]. Despite most patients developing mild symptoms, a small but significant group of patients will require intensive care unit (ICU) hospitalization and may present multiorgan dysfunction [2]. Knowledge about outcomes and short and long-term sequelae in this group of patients is still limited but is expected that survivors of critical illness may suffer from post-intensive care syndrome (PICS) and may maintain needs and symptoms after acute hospital discharge [3,4].

The sequelae after COVID-19 have become an increasing concern. Long-term persistent symptoms have been reported [5-7]. Sequelae persisting for longer than two months, usually three months from the onset of probable or confirmed SARS-CoV-2 infection, are included in the “post-COVID condition” [6]. Fatigue, persistent dyspnoea, exercise intolerance, and cognitive dysfunction are commonly reported symptoms, generally with an impact on everyday functioning [8,9].

In our previous study, we evaluated the impact of an inpatient multimodal and intensive rehabilitation program on neuromuscular, respiratory, and functional impairments of COVID-19 patients previously admitted to the ICU [4]. We found highly significant improvements with respect to neuromuscular, respiratory, and functional impairments.

The aim of this work was to reassess the functional capacity and respiratory status three months after discharge from an inpatient rehabilitation program.

## Materials and methods

Subjects and study design

This was a prospective study conducted in a specialized rehabilitation unit of neurological disorders in the North Rehabilitation Centre, Portugal, including patients previously hospitalized in the ICU due to SARS-Cov-2 pneumonia, consecutively admitted in the rehabilitation center between April 2020 and February 2021. Those patients were referred by an acute hospital physiatrist and those with 18 or more years and clinically stable were considered eligible to integrate an inpatient and intensive rehabilitation program. Those not previously admitted to the ICU, presenting major neurological syndromes, or still undergoing inpatient rehabilitation at the time of the study were excluded.

Forty-two post-ICU COVID-19 survivors completed an inpatient rehabilitation program. Six patients were excluded because they did not attend the three months follow-up consultation and tests. Hence, a total of 36 patients were included in this study.

Patients were evaluated at admission (T0), at discharge (T1), and three months after discharge (T2). The rehabilitation program protocol and the evaluations performed between T0 and T1 were already described by the authors elsewhere [4,10]. Three months after the discharge, all patients were called to a physical medicine and rehabilitation consultation and were asked to perform the tests in on an outpatient basis.

All patients gave their written informed consent prior to their inclusion in the study. All procedures performed were in accordance with ethical standards and the 1964 Helsinki Declaration and its later amendments.

Procedures and variables definition

Clinical data were collected by consulting the clinical file and interviewing the patient about sex, age, employment and marital status, comorbidities, and smoking habits. Acute hospital and ICU length of stay and respiratory support, as well as rehabilitation center length of stay, were considered. Lastly, information regarding the need for an outpatient rehabilitation program or the prescription of home-based physical exercises after discharge was also registered.

Clinical and functional assessments performed at T1 and T2 are described below.

Respiratory Function Assessment

Respiratory muscle strength: A manovacuometer (MicroRPM®, Carefusion, Basingstoke, UK) was used to measure the maximum inspiratory pressure (MIP) and maximum expiratory pressure (MEP), according to the American Thoracic Society/European Respiratory Society guidelines [11]. The maneuvers were performed three times and the best value was considered. The lower limit of normality of MIP and MEP was calculated considering age and sex [12].

Voluntary Cough Efficacy

Measurement of peak cough flow (PCF) (L/min) was performed with asmaPLAN+, Vitalograph Spirotrac 6800®, following the guidelines recommended by the American Thoracic Society/European Respiratory Society [11]. After three measurements, the highest value was considered. Cough was considered significantly reduced when CPF was <270 L/min and severely impaired when <160 L/min [13,14].

Functional Status Assessment

1 min sit-to-stand test (1STST):* *The 1STST has been accepted as an indicator of functional status and has been used to evaluate exercise tolerance and peripheral muscle strength [15]. The test was first demonstrated by a physiotherapist and then performed by the patient. The 1STST was performed with a standard height (46 cm) chair without armrests. The patient was seated upright on the chair with knees and hips flexed at 90°, feet placed flat on the floor a hip-width apart and arms held stationary by placing their hands on their hips. Patients were asked to perform repetitions of standing upright and then sitting down in the same position at a self-paced speed, as many times as possible for 1 min. The number of completed repetitions was recorded. The subjects were permitted to use rest periods to complete 1 min. Tests with a number of repetitions below the 2.5th percentile of population-based reference values, according to age and sex, were considered decreased [16].

6-minute walking test (6MWT): We conducted the 6MWT as described by the guidelines of the American Thoracic Society [17]. Subjects were instructed to walk in a 30-m indoor corridor at our center while attempting to cover as much distance as possible. They were allowed to stop during the test if necessary. A physiotherapist or nurse timed the walk and recorded the distance traveled, and at the end of the 6MWT, the total distance covered was recorded to the nearest meter. The percentage of predicted value was calculated using the reference equations developed by Enright and Sherrill [18]. Dyspnoea severity was assessed by the Modified Borg Scale (MBS) [19], heart rate (HR), blood pressure (BP), and pulsed oxygen saturation (SpO2) were measured before and at the end of tests.

Time up and go test (TUGT):* *The TUGT assesses the number of seconds needed for an individual to stand up from a chair, walk three meters at their usual pace, turn around, walk back to the chair, and sit down again. The score given is the time taken in seconds to complete the test [20]. Twelve seconds was considered the cut-off value in our sample, classifying normal vs. below-normal performance [21].

Brief-balance evaluation systems test (brief-BESTEST): The brief-BESTEST is a six-item balance test, which contains one item from each of the six subsections of the BESTEST. Each item is scored from 0 (severe balance impairment) to 3 (no balance impairment) and the maximum possible score is 24 points, with higher scores indicating better balance performance [22]. The brief-BESTEST was first demonstrated by a physiotherapist and then performed by the patient.

Fatigue: Fatigue was assessed using the Portuguese version of the fatigue assessment scale (FAS) by a physiotherapist. FAS measures the impact of fatigue on a patient´s activities and lifestyle [23]. Each item is scored on a Likert scale from 1 (“never”) to 5 (“always”) points, with a maximal score of 50 points, with higher scores implying worse fatigue.

Statistical methods

Continuous variables are expressed as means and standard deviations (SD) or medians with interquartile ranges for variables with skewed distributions. Categorical variables are presented as frequencies and percentages. Normal distribution was checked using the Shapiro-Wilk test or skewness and kurtosis.

Paired continuous variables comparisons were performed using paired student’s t-test or its non-parametric equivalent Wilcoxon test. Differences in categorical and continuous variables were tested with the use of Kruskal-Wallis or Mann-Whitney tests.

Pearson’s correlation coefficient (r) was used to assess the strength and direction of the linear relationships between pairs of continuous variables. Correlation coefficient values were interpreted as follows: 0.90 to 1.00 (-0.90 to -1.00): very high positive (negative) correlation; 0.70 to 0.90 (-0.70 to -0.90): high positive (negative) correlation; 0.50 to 0.70 (-0.50 to -0.70): moderate positive (negative) correlation; 0.30 to 0.50 (-0.30 to -0.50): low positive (negative) correlation; 0.00 to 0.30 (-0.00 to -0.30): negligible correlation.

Differences among the experimental groups were evaluated with the use of an analysis of variance (ANOVA) model, followed by the Tukey-Kramer test when findings with the ANOVA model were significant.

All reported p-values are two-tailed, with a p-value of 0.05 indicating statistical significance. Statistical analyses were performed using Software Statistical Package for the Social Sciences (SPSS) Version 23 (IBM Corp., Armonk, NY).

## Results

A total of 36 patients were included, with an average age of 62.49 ± 11.73 years. Demographic and clinical characteristics are summarized in Table 1. The comorbidities presented were prior to COVID-19 infection. After discharge, 20 patients (55.6%) maintained an outpatient rehabilitation program (including physiotherapy, and in some particular cases, also speech therapy); 11 patients (30.6%) performed home-based physical exercise, prescribed after discharge; three patients (8.3%) did not maintain any kind of rehabilitation program or home physical exercises; two patients (5.6%) lacked this information.

**Table 1:** Patients’ characteristics at baseline 1 – before acute hospitalization; a – body mass index ≥ 25kg/m2; b – peripheral arterial disease, chronic coronary syndrome, atrioventricular block, heart failure; c – asthma, obstructive sleep apnea, pulmonary emphysema, cured lung cancer; d – hypothyroidism; e –peptic ulcer, gastroesophageal reflux disease, chronic gastritis, nonalcoholic fatty liver disease; f – chronic kidney disease, benign prostatic hypertrophy, nephrolithiasis, cured urinary neoplasm, cured prostate cancer; g – osteoporosis, osteoarthritis, total knee arthroplasty, cruciate ligament injury; h – gout; i – anxiety, depression; j – benign paroxysmal positional vertigo, cured breast cancer

Sociodemographic variables	
Age (years)	62.49 ± 11.73
Sex – n (%)	
Male	29 (80.6)
Female	7 (19.4)
Employment status^1^ – n (%)	
Currently working	13 (36.1)
Medically discharged	7 (19.4)
Retired	15 (41.7)
Unemployed	1 (2.8)
Marital status – n (%)	
Single/ divorced/ widow	11 (30.6)
Married / civil union	25 (69.4)
Clinical variables	
Comorbidities – n (%)	
Cardiovascular risk factors	34 (94.4)
Hypertension	26 (72.2)
Type 1 or 2 diabetes mellitus	16 (44.4)
Overweight ^a^	19 (52.8)
Dyslipidemia	25 (69.4)
Cardiovascular ^b ^	7 (19.4)
Respiratory ^c ^	11 (30.6)
Endocrine ^d ^	5 (13.9)
Gastrointestinal ^e^	10 (27.8)
Genitourinary ^f ^	10 (27.8)
Musculoskeletal ^g^	5 (13.9)
Rheumatological ^h^	3 (8.3)
Psychiatric ^i^	1 (2.8)
Other ^j^	3 (8.3)
Smoking history - n (%)	
Current smoker	5 (13.9)
Never smoked	16 (44.4)
Previous smoker	15 (41.7)

The main acute hospitalization clinical details during COVID-19 infection and rehabilitation center settings are summarized in Table 2.

**Table 2:** Previous hospitalization clinical details ECMO – extracorporeal membrane oxygenation, HFOT – high flow oxygen therapy, ICU – intensive care unit, IV – invasive ventilation, FIM – functional independence measure, MRC - Medical Research Council

Acute hospital settings	
Acute hospital stay length (days) - median; IR	43.50; 30.00
ICU stay length (days) - mean ± SD	21.31 ± 11.10
Respiratory support - n (%)	
Invasive ventilation	30 (83.3)
HFOT	3 (8.3)
ECMO	3 (8.3)
Rehabilitation center settings	
Rehabilitation center stay length (days) - median; IR	30.00; 22.00
FIM at discharge - median; IR	
Motor	86.00;10.00
Cognitive	35.00;1.00
Total	120.00;7.00
Missing - n (%)	1 (2.8)
MRC at discharge - median; IR	58.00;4.80
< 48 – n (%)	1 (2.8)

A statistically significant difference was verified in MEP, PCF, 1’STST, 6-MWT, and FAS between T1 and T2 (Table 3) with higher values at T2 reflecting a significant clinical improvement in all these evaluations but FAS. On the contrary, no differences were seen in MIP, brief-BESTEST, or TUG. All patients presented a MIP above the lower limit of normality, but four patients maintained an MEP below the lower limit of normality, according to age and sex.

**Table 3:** Analysis of respiratory status and neuropsychological and functional assessment at the end of the rehabilitation program and at the three-month follow-up MIP – maximum inspiratory pressure, MEP – maximum expiratory pressure, PCF – peak cough flow, Brief-BESTEST – brief balance evaluation systems test, TUG – timed up and go, 1’STST – 1-minute sit to stand test, 6-MWT – 6-minute walking test, FAS – fatigue assessment scale, LLN – lower limit of normality

	T1	T2	Δ T2 - T1
Respiratory			
MIP (cmH2O) - mean ± SD	77.57 ± 22.92	81.77 ± 24.48	P = 0.168
< LLN – n (%)	1 (2.8)	0	
≥ LLN – n (%)	31 (86.1)	32 (88.9)	
Missings	4 (11.1)	2 (5.6)	
MEP (cmH2O) - mean ± SD	84.47 ± 20.89	97.23 ± 24.63	P < 0.001 Δ = 21.43 ± 23.10
< LLN – n (%)	5 (13.9)	4 (11.1)	
≥ LLN – n (%)	27 (75.0)	30 (83.3)	
Missings	4 (11.1)	2 (5.6)	
PCF (L/min) - mean ± SD	367.83 ± 117.24	441.33 ± 132.90	P = 0.003 Δ = 59.29 ± 114.76
< 160 – n (%)	1 (2.8)	1 (2.8)	
160 – 269 – n (%)	6 (16.7)	1 (2.8)	
≥ 270 – n (%)	25 (69.4)	32 (88.9)	
Missing – n (%)	4 (11.1)	2 (5.6)	
Functional			
Brief-BESTEST - mean ± SD	16.52 ± 4.49	17.68 ± 3.66	P = 0.079
Missing – n (%)	4 (11.1)	2 (5.6)	
TUG (seconds) - median; IR	9.00;2.30	8.50;3.00	P = 0.558
0 points – n (%)	0	0	
< 12 seconds – n (%)	26 (72.2)	27 (75.0)	
≥12 seconds – n (%)	6 (16.7)	7 (19.4)	
Missing– n (%)	4 (11.1)	2 (5.6)	
1’ STST - mean ± SD	19.90 ± 6.37	23.13 ± 6.07	P = 0.004 Δ = 2.71 ± 6.07
< reference value– n (%)	13 (36.1)	10 (27.8)	
Missing – n (%)	4 (11.1)	2 (5.6)	
6-MWT (meters) - mean ± SD	459.25 ± 153.70	500.00 ± 163.74	P = 0.003 Δ = 32.71 ± 27.60
% Predicted value	77.10 ± 21.85	88.06 ± 26.11	P = 0.006 Δ = 9.93 ± 7.96
Missing – n (%)	8 (22.2)	7 (19.4)	
FAS - median; IR	19.00;4.30	21.50;5.00	P = 0.002
Missing – n (%)	4 (11.1)	2 (5.6)	

When analyzing correlations between the T1-T2 variations of MEP, PCF, 1’STST, 6-MWT, and FAS, with age, acute hospital LOS, ICU LOS, rehabilitation LOS, age, MRC score, and FIM at discharge, only a low positive correlation was found between 1’STST and acute hospital LOS (Table 4).

**Table 4:** Correlations between respiratory and functional variations; and hospitalization and rehabilitation LOS, age, muscle strength, and functional status at discharge MEP – maximum expiratory pressure (cmH2O), PCF – peak cough flow (L/min), 1’ STST – 1 minute sit to stand test, 6-MWT – 6-minute walking test, FAS – fatigue assessment scale, LOS – length of stay, ICU – intensive care unit, MRC - Medical Research Council, FIM – functional independence measure

	Age	Acute hospital LOS	ICU LOS	Rehabilitation centre LOS	T1 score MRC	T1 FIM Total
Δ MEP	p = 0.145	p = 0.137	p = 0.628	p = 0.438	p = 0.120	p= 0.603
Δ PCF	p =0.794	P = 0.481	p = 0.751	p = 0.470	p = 0.741	p= 0.248
Δ 1´-STST	p = 0.515	ρ = 0.397	p = 0.268	p = 0.646	p = 0.135	p= 0.268
		p = 0.027				
Δ 6MWT	p = 0.748	p = 0.682	p = 0.724	p = 0.565	p= 0.920	p= 0.996
Δ FAS	p = 0.819	p = 0.561	p = 0.270	p = 0.086	p= 0.746	p= 0.270

There was no statistically significant difference between the type of rehabilitation needs after discharge (outpatient rehabilitation program or prescribed home physical exercises) with the T1-T2 variations of MEP (p=0.192), PCF (p=0.057), 1’STST (p=0.376), 6-MWT (p=0.647), and FAS (p=0.493).

## Discussion

Three months after discharge, patients did not only maintain the gains of the previous inpatient rehabilitation but also showed an improvement in MEP and PCF. However, MEP and PCF variation did not correlate with any of the analyzed variables.

There was no statistically significant difference in MIP values between T1 and T2, which may be due to a ceiling effect since only one patient presented a MIP value below the lower limit of normality at discharge. In addition, all patients presented a MIP above the lower limit of normality.

Hennigs JK et al. [9], in a small cross-sectional pilot study of mild-to-moderate and moderate-to-critical COVID-19 convalescent patients, found inspiratory muscle strength decreased below sex and age-specific cutoffs in 88% of patients, five months after acute infection. Although this impairment was predominantly in patients previously hospitalized due to COVID-19, inspiratory muscle weakness also occurred frequently in non-hospitalized patients (65%). As compared to Hennigs JK et al. [9], this did not mention the enrolment in a rehabilitation program; our results are quite different and may point toward the importance of rehabilitation and intervention in respiratory muscle strength. The different PIM determination using the peak value of maximum inspiratory mouth pressure measured from residual volume may also justify partially some of the differences found. The presence of only 19.4% of women in our population may also underestimate our results since Hennins JK et al. [9] found that inspiratory muscle weakness was more frequent in women.

Despite these encouraging results, four patients (11.1%) maintained MEP below the lower limit of normality. Anastasio F et al. found a predicted mean MEP value of 64% in patients who were submitted to invasive mechanical ventilation four months after SARS-CoV-2 diagnosis with pneumonia [24]. The authors suggest that decreased MEP could be explained by the combination of multiple factors such as myopathy, curarization, corticosteroids, and a temporary lack of spontaneous respiratory movements [24].

Huang et al. evaluated 57 patients 30 days after discharge of acute hospital stay due to COVID-19, not specifying the necessity of ICU hospitalization [25]. In this study, more than half of the subjects had impairment of respiratory muscle strength, and 22.8% presented MEP values of less than 80% of the predicted value. Comparatively, our reassessment was performed later and after an inpatient rehabilitation program and may simultaneously be the result of a progressive improvement of expiratory muscle strength or reflect the benefits of rehabilitation.

Regarding functional capacity, a significant walking distance improvement in the 6MWT was verified, with mean predicted values of 88.06 ± 26.11% three months after discharge. Huang C et al. found similar values in patients discharged from the hospital and evaluated six months later - 495.0 (450.0 - 540.0) meters, 88% (79.5 - 96.4) [7]. However, only 4% of them were admitted to the ICU, reflecting a less severe disease and a different population. On the other hand, in Anastasio F. et al.'s study including COVID-19 patients who needed invasive ventilation, this value was somewhat lower (70% of predicted distance) four months after SARS-CoV-2 diagnosis, in an outpatient visit [24]. However, in both of these works, enrolment in a rehabilitation program was not considered. This fact may compromise the comparison of results with our population since the functional improvement may again simultaneously be the result of a natural and progressive improvement or reflect the benefits of rehabilitation.

However, long-term exercise limitations, as well as physical and psychological sequelae, in patients that suffered acute respiratory distress syndrome (ARDS) and in critical illness survivors may be expected [26]. In a five-year follow-up, ARDS survivors maintained a persistent reduction in their ability to exercise [26].

We also noted a significant difference in median STST between T1 and T2, reinforcing functional capacity improvement. Longer acute hospital stay length was associated with greater improvement in Brief-BESTest. Longer acute hospital length of stay may be associated with a more severe and prolonged acute disease and hence with a lower performance at baseline.

However, there was no improvement in the TUG test. About 19.4% of patients maintained a score above the cut-off considered, suggesting functional and mobility impairment [23]. This test has been used to assess functional mobility, walking ability, dynamic balance, and risk of falling in subjects with a variety of conditions [27,28], and may indicate the maintenance of rehabilitation needs.

We found no difference in brief-BESTEST performance at the three-month follow-up. This may be explained due to a ceiling effect since all patients improved their performance at the end of the inpatient rehabilitation program.

Despite the overall improvement of functional capacity, our sample reported higher scores of FAS, indicating worsening fatigue levels after three months. However, T2 FAS values still belong to the range of values indicating non-fatigued persons [23,28]. Fatigue has increasingly been reported by COVID-19 patients with previous moderate to severe disease, achieving up to 70% of prevalence in some series [24,29]. However, according to the recent review of Sandler CX et al., most COVID-19 cohort studies report persistent fatigue ranging from 13% to 33% at 16 to 20 weeks post-symptom onset [30]. Despite fatigue being reported as a dominant complaint in “long COVID,” it should be noted that it is a multifactorial and subjective symptom, involving biological and psychological aspects, with physiopathology not completely understood. Since there was an overall improvement in functional status, it would be interesting to evaluate the emotional status and its correlation with fatigue. Larger prospective studies with longer follow-ups, using more comprehensive methods for the assessment of fatigue and related conditions, are needed.

After discharge, 31 patients (86.2%) maintained some kind of rehabilitation needs, including an outpatient rehabilitation program or home-based physical exercise, prescribed after discharge. However, we did not find any difference between the type of rehabilitation needs after discharge (outpatient rehabilitation program or prescribed home physical exercises) with T1-T2 variations of MEP, PCF, 1’STST, 6-MWT, and FAS. The small sample may have compromised any significant relation.

This study presents some limitations. First, our sample may not be representative of the post-ICU COVID-19 patient population due to a selection bias since our rehabilitation center receives only the more severe cases. Second, due to the absence of a control group, it is not possible to report on the causality of the observed conclusions. Last, information regarding levels of physical activity after discharge was not considered, not allowing interesting relations and considerations. Also, a three-month follow-up may not be enough to understand the clinical evolution of these patients in the long term and the persistence of sequelae in the future.

This three-month follow-up reinforces the importance and actual necessity of monitoring these patients after discharge from an inpatient rehabilitation program, to investigate and optimize the clinical intervention.

## Conclusions

Post-ICU COVID-19 survivors admitted to an inpatient rehabilitation program maintained good functional recovery at the three-month follow-up. There was also improvement in respiratory muscle and cough strength. These findings reinforce the importance of rehabilitation in this population. Despite overall improvement, we found higher scores of FAS, suggesting worse fatigue levels. Clinical and instrumental long-term evaluations of these patients are advisable, allowing timely intervention, particularly in a rehabilitation program.
